# Supplementary material for: A fault-intrusion-tolerant system and deadline-aware algorithm for scheduling scientific workflow in the cloud
Source: PeerJ Comput Sci. 2021 Nov 2;7:e747. doi: 10.7717/peerj-cs.747 (PMC8576554; doi:10.7717/peerj-cs.747)
Supplement: Supplemental Information 6 [file peerj-cs-07-747-s006.zip › Myproject1.1/docs/org/cloudbus/cloudsim/provisioners/class-use/RamProvisioner.html]

Uses of Class org.cloudbus.cloudsim.provisioners.RamProvisioner


JavaScript is disabled on your browser.


- Overview
- Package
- Class
- Use
- Tree
- Deprecated
- Index
- Help

- Prev
- Next

- Frames
- No Frames

- All Classes

## Uses of Class org.cloudbus.cloudsim.provisioners.RamProvisioner

- Packages that use RamProvisioner

  | Package | Description |
  | --- | --- |
  |  |  |
  | --- | --- |
  | org.cloudbus.cloudsim |  |
  | org.cloudbus.cloudsim.network.datacenter |  |
  | org.cloudbus.cloudsim.power |  |
  | org.cloudbus.cloudsim.provisioners |  |
- - ### Uses of RamProvisioner in org.cloudbus.cloudsim

    Methods in org.cloudbus.cloudsim that return RamProvisioner

    | Modifier and Type | Method and Description |
    | --- | --- |
    |  |  |
    | --- | --- |
    | `RamProvisioner` | Host.`getRamProvisioner()` Gets the ram provisioner. |

    Methods in org.cloudbus.cloudsim with parameters of type RamProvisioner

    | Modifier and Type | Method and Description |
    | --- | --- |
    |  |  |
    | --- | --- |
    | `protected void` | Host.`setRamProvisioner(RamProvisioner ramProvisioner)` Sets the ram provisioner. |

    Constructors in org.cloudbus.cloudsim with parameters of type RamProvisioner

    Constructor and Description

    `Host(int id,
    RamProvisioner ramProvisioner,
    BwProvisioner bwProvisioner,
    long storage,
    java.util.List<? extends Pe> peList,
    VmScheduler vmScheduler)`

    Instantiates a new host.

    `HostDynamicWorkload(int id,
    RamProvisioner ramProvisioner,
    BwProvisioner bwProvisioner,
    long storage,
    java.util.List<? extends Pe> peList,
    VmScheduler vmScheduler)`

    Instantiates a new host.
  - ### Uses of RamProvisioner in org.cloudbus.cloudsim.network.datacenter

    Constructors in org.cloudbus.cloudsim.network.datacenter with parameters of type RamProvisioner

    Constructor and Description

    `NetworkHost(int id,
    RamProvisioner ramProvisioner,
    BwProvisioner bwProvisioner,
    long storage,
    java.util.List<? extends Pe> peList,
    VmScheduler vmScheduler)`
  - ### Uses of RamProvisioner in org.cloudbus.cloudsim.power

    Constructors in org.cloudbus.cloudsim.power with parameters of type RamProvisioner

    Constructor and Description

    `PowerHost(int id,
    RamProvisioner ramProvisioner,
    BwProvisioner bwProvisioner,
    long storage,
    java.util.List<? extends Pe> peList,
    VmScheduler vmScheduler,
    PowerModel powerModel)`

    Instantiates a new host.

    `PowerHostUtilizationHistory(int id,
    RamProvisioner ramProvisioner,
    BwProvisioner bwProvisioner,
    long storage,
    java.util.List<? extends Pe> peList,
    VmScheduler vmScheduler,
    PowerModel powerModel)`

    Instantiates a new power host utilization history.
  - ### Uses of RamProvisioner in org.cloudbus.cloudsim.provisioners

    Subclasses of RamProvisioner in org.cloudbus.cloudsim.provisioners

    | Modifier and Type | Class and Description |
    | --- | --- |
    |  |  |
    | --- | --- |
    | `class` | `RamProvisionerSimple` RamProvisionerSimple is an extension of RamProvisioner which uses a best-effort policy to allocate memory to a VM. |

- Overview
- Package
- Class
- Use
- Tree
- Deprecated
- Index
- Help

- Prev
- Next

- Frames
- No Frames

- All Classes
